# Personalized treatment hierarchies in Bayesian network meta-analysis

**DOI:** 10.1017/rsm.2026.10089

**Published:** 2026-05-06

**Authors:** Augustine Wigle, Erica E. M. Moodie

**Affiliations:** Epidemiology, Biostatistics and Occupational Health, McGill University, Canada

## Abstract

Network meta-analysis (NMA) is an increasingly popular evidence synthesis tool that can provide a ranking of competing treatments, also known as a treatment hierarchy. Treatment–covariate interactions (TCIs) can be included in NMA models to allow relative treatment effects to vary with covariate values. We show that in an NMA model that includes TCIs, treatment hierarchies should be created with a particular covariate profile in mind. We outline the typical approach for creating a treatment hierarchy in standard Bayesian NMA and show how a treatment hierarchy for a particular covariate profile can be created from an NMA model that estimates TCIs. We demonstrate our methods using a real network of studies for the treatment of major depressive disorder.

## Highlights

### What is already known?

- Network meta-analyses (NMAs) that include treatment–covariate interactions, such as individual participant data (IPD) NMAs, provide relative treatment effects conditional on covariate values.
- Treatment hierarchies created from ranking metrics are a popular way to summarize the results of a network meta-analysis.

### What is new?

- We describe how existing ranking metrics can be easily adapted to provide personalized treatment hierarchies from an IPD NMA.

### Potential impact for RSM readers

- Practitioners should consider creating tools that can compute ranking metrics in IPD NMAs to support personalized clinical decision-making.

## 1 Introduction

Network meta-analysis (NMA) is a statistical methodology that facilitates the estimation of relative effects for multiple treatments from a set of clinical trials. A convenient method for summarizing the results of a standard NMA is to construct a treatment hierarchy, that is, a list of the treatments ranked in order of preference. Treatment hierarchies are constructed using ranking metrics that consider the magnitude and uncertainty of relative effect estimates, such as the Surface Under the Cumulative RAnking curve (SUCRA) in Bayesian NMA.^1^^,^ ^2^ A treatment hierarchy is a desired output of many NMAs.^3^

When effect modifiers are present, incorporating treatment–covariate interactions (TCIs) into the NMA model can reduce heterogeneity and provide more precise estimates.^4^^–^ ^6^ TCIs allow the expected relative treatment effects, and therefore treatment hierarchies, to differ between individuals with different covariate values, presenting an opportunity to compute personalized hierarchies. Authors have called for the reporting of treatment hierarchies at different covariate levels when TCIs are included in an NMA,^4^^–^ ^7^ but this is rarely done in practice. In this article, we show how Bayesian NMAs with TCIs can produce personalized treatment hierarchies, and demonstrate the methods using a network of studies for major depressive disorder (MDD).

## 2 Treatment hierarchies in standard NMA models

In a standard NMA with no TCIs, the parameters representing treatment performance are given by $\psi_{20}^{\left(1\right)},\psi_{30}^{\left(1\right)},\ldots ,\psi_{G0}^{\left(1\right)}$ , where $\psi_{g0}^{\left(1\right)}$ is the expected relative effect of treatment *g* compared to treatment 1, and the index 0 indicates that the parameter represents the main effect of the treatment and not an interaction, which will become important in the following section. Additionally, *G* is the number of treatments in the network, and $\psi_{10}^{\left(1\right)}=0$ . We assume that the expected relative effect of treatment *g* versus *h*, $\psi_{g0}^{\left(h\right)}$ , is given by $\psi_{g0}^{\left(1\right)}-\psi_{h0}^{\left(1\right)}$ for any g,h in 1,…,G . This is referred to as the consistency assumption in NMA literature.^8^ In the remainder of the article, we suppress the superscript (1) and emphasize that all parameters are relative to treatment 1.

SUCRA values are calculated from the relative effects to obtain a treatment hierarchy that is relevant to the population represented by the NMA. The SUCRA value for treatment *g* is calculated in a standard Bayesian NMA as

(2.1) $$\begin{array}{rl} \text{SUCRA}(g) & =\frac{\sum_{s=1}^{G-1}\sum_{r=1}^{s}p_{gr}}{G-1}\\ & =\frac{G-\text{E}\left[rank(g)\right]}{G-1}, \end{array}$$

where $p_{gr}$ is the probability that treatment *g* takes rank *r*, that is, it has a more favorable response than exactly *r* treatments, and E[rank(g)] is the average rank of treatment *g*.^2^^,^ ^9^

SUCRA values can be computed in a standard Bayesian NMA using samples from the posterior distribution. Assume that we have a sample of size *M* from the posterior distribution of $\psi_{10}=0,\psi_{20},\ldots ,\psi_{G0}$ , and let $\psi_{g0}{}_{\left(m\right)}$ represent the value of $\psi_{g0}$ in the $m^{\text{th}}$ posterior sample. Note that $\psi_{10}=0$ by definition for every posterior sample. Then we can calculate SUCRA values as follows:

1. For each sample m=1,…,M and each treatment g=1,…,G , determine the treatment rank according to
$$\begin{array}{r} rank(g{)}_{\left(m\right)}=G-\sum_{h\neq g}I\left\{\psi_{g0}{}_{\left(m\right)}>\psi_{h0}{}_{\left(m\right)}\right\}, \end{array}$$
  where I{⋅}=1 when ⋅ is true and 0 otherwise.
2. Calculate the average rank for each treatment,
$$\begin{array}{r} \text{E}\left[rank(g)\right]=\frac{1}{M}\sum_{m=1}^{M}rank(g{)}_{\left(m\right)}. \end{array}$$
3. Calculate SUCRA(g) using
$$\begin{array}{r} \text{SUCRA}(g)=\frac{G-\text{E}\left[rank(g)\right]}{G-1}. \end{array}$$

The SUCRA of treatment *g* in a standard NMA is interpreted as the proportion of competing treatments that *g* is expected to beat,^2^ or the inverse-scaled average rank of treatment *g*,^9^ *when it is employed in the population represented by the NMA*. The resulting treatment hierarchy is not personalized to any particular patient and depends on the covariate distribution in the population.^10^

## 3 Treatment hierarchies in NMA models with TCIs

TCIs can be estimated using aggregate data NMA models with meta-regression and Individual Participant Data (IPD) NMA models, including recently proposed Individualized Treatment Rule (ITR) NMA models.^11^^,^ ^12^ In Supplementary Section S1, we describe the suitability of each model type for creating personalized treatment hierarchies. Briefly, we do not recommend using aggregate data NMA meta-regression to create personalized treatment hierarchies due to its risk of ecological bias and confounding.^11^^,^ ^13^^–^ ^15^ IPD NMA can be used to produce personalized treatment hierarchies when care is taken in specifying the model.^6^^,^ ^7^^,^ ^16^

In an NMA model that includes TCIs, the interpretation of the parameter $\psi_{g0}$ does not correspond to the expected relative effect on average in the population. Instead, $\psi_{g0}$ corresponds to the expected relative effect of treatment *g* compared to treatment 1 in the reference group. To illustrate, suppose there are *Q* effect-modifying covariates included as TCIs in an NMA model. The expected relative effect of treatment *g* versus treatment 1 for an individual with covariate values $X_{1}=x_{1},X_{2}=x_{2},\ldots ,X_{Q}=x_{Q}$ is given by

(3.1) $$\begin{array}{r} \psi_{g0}+\psi_{g1}x_{1}+\psi_{g2}x_{2}+\cdots +\psi_{gQ}x_{Q}, \end{array}$$

where $\psi_{g1},\ldots ,\psi_{gQ}$ are the coefficients representing the interaction between each covariate and treatment *g* compared to treatment 1, that is, $\psi_{gq},q>0$ is the change in the relative effect of treatment *g* compared to treatment 1 for a one unit increase in covariate *q*, and $\psi_{g0}$ is the expected relative effect when all $x_{1},\ldots ,x_{Q}$ equal 0.

Now, consider creating a treatment hierarchy from a model that includes TCIs. If we were to use only $\psi_{g0}$ , g=1,…,G to create the hierarchy for such a model, the hierarchy would apply to patients whose covariate values are all equal to zero. The hierarchy will be meaningless for patients with a different covariate profile. Clearly, for a treatment hierarchy to be meaningful in an NMA with TCIs, it should be intentionally created for a particular set of covariate values.

Let $\text{E}\left[rank(g)|x_{1},\ldots ,x_{Q}\right]$ be the average rank of treatment *g* given covariates $x_{1},\ldots ,x_{Q}$ . The average ranks are determined using the expected relative effects defined in equation (3.1). They can be calculated using samples from the posterior of $\psi_{gq}$ , g=1,… , *G* and q=0 , … , *Q*

1. For each sample m=1,…,M and each treatment g=1,…,G , determine the expected relative treatment effect given $x_{1},\ldots ,x_{Q}$ via
$$\begin{array}{r} RE_{1g}{}_{\left(m\right)}(x_{1},\ldots ,x_{Q})=\psi_{g0}{}_{\left(m\right)}+\psi_{g1}{}_{\left(m\right)}x_{1}+\psi_{g2}{}_{\left(m\right)}x_{2}+\cdots +\psi_{gQ}{}_{\left(m\right)}x_{Q}\;\text{for }g=2,...,G, \end{array}$$
  and $RE_{11}{}_{\left(m\right)}(x_{1},\ldots ,x_{Q})=0$ , where $RE_{1g}{}_{\left(m\right)}(x_{1},\ldots ,x_{Q})$ represents the expected relative effect of treatment *g* versus 1 given covariates $x_{1},\ldots ,x_{Q}$ in sample *m*.
2. For each sample m=1,…,M and each treatment g=1,…,G , determine the treatment rank according to
$$\begin{array}{r} rank(g{)}_{\left(m\right)}=G-\sum_{h\neq g}I\left\{RE_{1g}{}_{\left(m\right)}(x_{1},\ldots ,x_{Q})>RE_{1h}{}_{\left(m\right)}(x_{1},\ldots ,x_{Q})\right\}. \end{array}$$
3. Calculate the average rank for each treatment given the covariates,
$$\begin{array}{r} \text{E}\left[rank(g)|x_{1},\ldots ,x_{Q}\right]=\frac{1}{M}\sum_{m=1}^{M}rank(g{)}_{\left(m\right)}. \end{array}$$
4. Calculate SUCRA(g) using
$$\begin{array}{r} \text{SUCRA}(g)=\frac{G-\text{E}\left[rank(g)|x_{1},\ldots ,x_{Q}\right]}{G-1}. \end{array}$$

The treatment hierarchy produced in this way is personalized to a patient with the covariate profile defined by $x_{1},\ldots ,x_{Q}$ .

## 4 Example: Pharmacological treatments for MDD

We illustrate the use and impact of personalized treatment hierarchies using data from three studies investigating treatment for MDD. A systematic review process was not followed to select the studies; rather, the studies were selected based on the availability of IPD. Details on the study designs are given in Supplementary Section S2. The data provide information on six pharmacological treatments: Bupropion, Citalopram + Bupropion, Citalopram + Buspirone, Escitalopram, Sertraline, and Venlafaxine. The response is defined as the negative of the 17-question Hamilton Rating Scale for Depression (HRSD-17), so that larger values are preferred. The following covariates, considered to be potential effect modifiers for depression, were included as TCIs in the analysis: age, sex, marital status, years of education, employment status, household size, age of depression onset, number of depressive episodes, chronicity of current MDD episode, and HRSD-17 before receiving any treatment in the study.^17^^,^ ^18^ We fit a two-stage common effect Bayesian ITR NMA model with IPD to estimate expected relative treatment effects in the MDD network,^12^ described in detail in Supplementary Section S3.

We computed personalized treatment hierarchies using SUCRA for two hypothetical patients, Patient A and Patient B, each with unique covariate profiles: Patient A is employed while Patient B is unemployed, Patient A has experienced three or fewer depressive episodes while Patient B has experienced more than three, Patient A is male and Patient B is not, and Patient A lives in a household with two more members than Patient B. The specific covariate values for each patient are described in Supplementary Section S3.3.

### 4.1 Results

**Figure 1:**
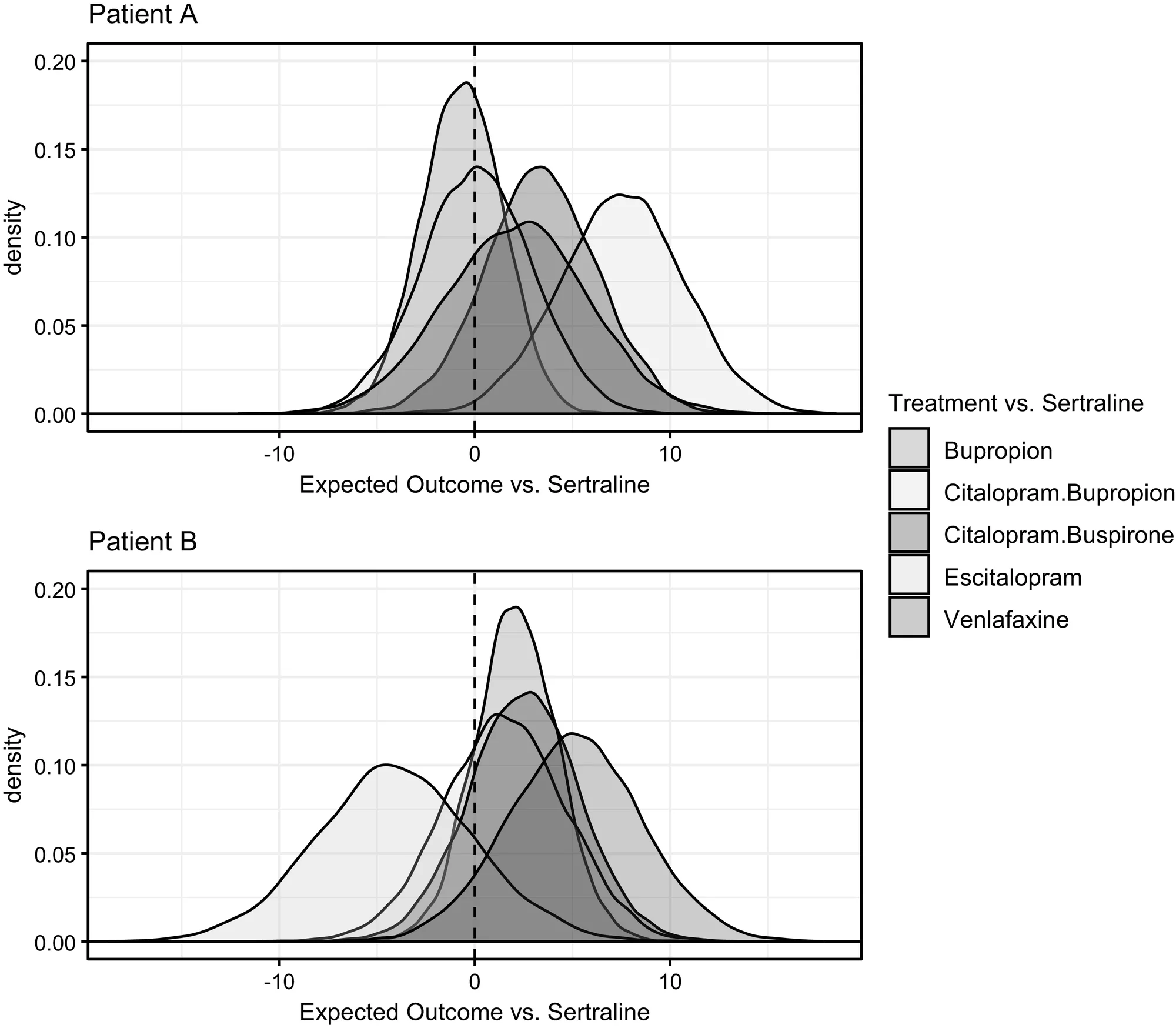
Posterior distributions of the relative effects of each treatment for Patients A and B. Figure 1 Long description.

A plot showing point estimates and 95% credible intervals for treatment effects and TCIs is shown in Supplementary Section S3.4. All credible intervals contain zero. This is not too surprising, since only three studies were included in the analysis. In Figure 1, we show the posterior distribution of the expected relative effects as defined in equation (3.1) of each treatment compared to Sertraline for Patients A and B. It is clear that there are differences in the posterior densities between the patients. The SUCRA values for Patients A and B reflect the differences in the posteriors, and are compared in Table 1. For example, Citalopram + Bupropion is ranked first for Patient A, while it is ranked only fourth out of the six treatments for Patient B. Table 1 also compares the SUCRA and treatment rankings that would result from using a naive method that only considered the main treatment effects, $\psi_{g0}$ . The naive method gives the same hierarchy for both patients, and this differs from both personalized hierarchies, particularly for Patient B, who deviates more from the reference profile.

**Table 1:** SUCRA values and ranking of each treatment using a naive nonpersonalized approach, and using a personalized approach for Patient A and Patient B Table 1 Long description.

Treatment	Naive		Patient A		Patient B	
	SUCRA	Rank	SUCRA	Rank	SUCRA	Rank
Bupropion	0.30	5	0.22	6	0.59	3
Citalopram+Bupropion	0.81	1	0.96	1	0.50	4
Citalopram+Buspirone	0.79	2	0.66	2	0.63	2
Escitalopram	0.20	6	0.33	4	0.10	6
Venlafaxine	0.53	3	0.52	3	0.87	1
Sertraline	0.38	4	0.30	5	0.31	5

## 5 Discussion

Personalized treatment hierarchies can be computed from IPD NMA models that include TCIs. We demonstrated the methods using an ITR NMA of treatments for MDD, where the TCIs were selected based on previous studies on MDD effect modifiers. The posterior distributions of the expected relative effects changed for different patient profiles and resulted in different treatment hierarchies, suggesting possible value in personalizing recommendations.

We caution against relying on the statistical significance of TCI parameters to decide when it would be beneficial to create personalized treatment hierarchies. First, TCIs that exist in practice but have not yet been estimated precisely enough to reach statistical significance are still important to include in the model, and a personalized treatment hierarchy could be useful in this context. Additionally, although the $\psi_{gq}$ parameters may not be statistically significantly different from zero, these parameters represent interactions compared to the reference treatment, and it is possible that other interaction terms may be statistically significant for other treatment comparisons, for example, the credible interval for the interaction of age with Bupropion versus Sertraline may contain zero, but the credible interval for the interaction of age with Bupropion versus Venlafaxine may be well away from zero. Therefore, an interaction that does not seem important at first glance may indeed be important for other treatment comparisons.

On the other hand, including TCIs that do not exist in practice will increase the variance of the posterior distribution of conditional relative effects without improving the accuracy of treatment recommendations. The use of the SUCRA ranking metric to create the hierarchy will mitigate the additional variance to some extent, as SUCRA summarizes the entire posterior distribution of relative effects,^19^ but personalized treatment hierarchies may still be misleading if the included TCIs do not exist. For this reason, we believe personalized treatment hierarchies should be created when there is additional reason to believe that the included TCIs do exist in practice, besides only the NMA results at hand.

We focused on creating treatment hierarchies from the expected relative effects. In random-effects NMA models, treatment effects and interactions are allowed to vary across studies, and a personalized treatment hierarchy that better reflects uncertainty could be obtained by considering this additional predictive variability. A Bayesian approach to creating predictive treatment hierarchies in standard NMAs has been proposed^20^ and could be adapted to personalized treatment hierarchies.

We echo previous calls to report treatment hierarchies for different covariate profiles when possible and when there is evidence that TCIs exist.^4^^–^ ^7^ If many covariates are effect modifiers, it may not be feasible to report hierarchies for all covariate patterns of interest. Some recent IPD NMAs have included online tools that allow individuals to compute expected relative effects for custom covariate values to aid in decision-making.^21^^,^ ^22^ With little additional effort, personalized treatment hierarchies could be included in the output of such tools. We encourage researchers conducting IPD NMAs to consider creating tools that can provide personalized treatment hierarchies to support clinical decision-making.

## Supporting information

10.1017/rsm.2026.10089.sm001 Wigle and Moodie supplementary material Wigle and Moodie supplementary material

## Data Availability

Code and data to reproduce the results in this article are available at https://doi.org/10.5281/zenodo.19007228.

## Supplementary material

To view supplementary material for this article, please visit http://doi.org/10.1017/rsm.2026.10089.

## Long descriptions

### Table 1 Long description

The table consists of seven columns: Treatment, Naive S U C R A, Naive Rank, Patient A S U C R A, Patient A Rank, Patient B S U C R A, and Patient B Rank.

* Bupropion: Naive 0.30 (Rank 5); Patient A 0.22 (Rank 6); Patient B 0.59 (Rank 3).

* Citalopram plus Bupropion: Naive 0.81 (Rank 1); Patient A 0.96 (Rank 1); Patient B 0.50 (Rank 4).

* Citalopram plus Buspirone: Naive 0.79 (Rank 2); Patient A 0.66 (Rank 2); Patient B 0.63 (Rank 2).

* Escitalopram: Naive 0.20 (Rank 6); Patient A 0.33 (Rank 4); Patient B 0.10 (Rank 6).

* Venlafaxine: Naive 0.53 (Rank 3); Patient A 0.52 (Rank 3); Patient B 0.87 (Rank 1).

* Sertraline: Naive 0.38 (Rank 4); Patient A 0.30 (Rank 5); Patient B 0.31 (Rank 5).

Navigate back to Table 1.

### Figure 1 Long description

The figure consists of two vertically stacked density plots. Both plots share a y-axis labeled density ranging from 0.00 to 0.20 and an x-axis labeled Expected Outcome vs. Sertraline ranging from -10 to 10. A vertical dashed line at 0 represents the baseline effect of Sertraline.

Top panel, Patient A:

* Bupropion (pink) has a high peak centered slightly below 0.

* Escitalopram (blue) has a broad, low peak centered around 2.

* Citalopram.Buspirone (green) has a peak centered around 4.

* Venlafaxine (purple) has a peak centered around 5.

* Citalopram.Bupropion (yellow) has a peak centered furthest to the right, around 8.

Bottom panel, Patient B:

* Escitalopram (blue) is the only treatment with a peak significantly to the left of the baseline, centered around -5.

* Bupropion (pink) has the highest peak, centered slightly above 0.

* Citalopram.Buspirone (green) and Citalopram.Bupropion (yellow) have overlapping peaks centered between 0 and 5.

* Venlafaxine (purple) has a peak centered around 6.

A legend to the right identifies the five treatments: Bupropion (pink), Citalopram.Bupropion (yellow), Citalopram.Buspirone (green), Escitalopram (blue), and Venlafaxine (purple).

Navigate back to Figure 1.
