# Personalized treatment hierarchies in Bayesian network meta-analysis – ADDENDUM

**DOI:** 10.1017/rsm.2026.10105

**Published:** 2026-06-24

**Authors:** Augustine Wigle, Erica E. M. Moodie

This manuscript was originally published with acknowledgements section omitted from the back matter. The correct acknowledgements information can be found below:

Acknowledgements. Data and/or research tools used in the preparation of this manuscript were obtained from the National Institute of Mental Health (NIMH) Data Archive (NDA). NDA is a collaborative informatics system created by the National Institutes of Health to provide a national resource to support and accelerate research in mental health. Dataset identifier(s): #2199 (EMBARC), #2153 (REVAMP), #2148 (STAR*D). This manuscript reflects the views of the authors and may not reflect the opinions or views of the NIH or of the Submitters submitting original data to NDA.

This has now been added to the original manuscript.
