# Supplementary material for: Personalized treatment hierarchies in Bayesian network meta-analysis
Source: Res Synth Methods. 2026 May 6;17(5):1018–24. doi: 10.1017/rsm.2026.10089 (PMC13732360; doi:10.1017/rsm.2026.10089)
Supplement: Supplementary file 1 [file S1759287926100891sup001.pdf]

Supplementary Information for
Personalized Treatment Hierarchies in Bayesian Network Meta-Analysis

Augustine Wigle   Erica E. M. Moodie

S1 Overview of NMA Models for Estimating Treatment-Covariate Interactions

S1.1 Aggregate Data NMA with Meta-Regression

When study-level aggregated covariate data are available, such as the average age or the proportion of patients with severe disease in the study, TCIs can be included in the NMA model via meta-regression. Interactions are estimated using only across-trial information in these models [1, 2]. Interactions estimated using across-trial information are subject to ecological bias, meaning that across-trial interactions can differ from the true patient-level interactions, as well as confounding, since covariates are balanced by randomization within studies, but not across studies [2, 3, 4]. Due to the risk of bias, using an aggregate data NMA meta-regression to produce a personalized treatment hierarchy is not recommended.

S1.2 Individual Participant Data NMAs

When outcome and covariate data at the patient level are available, an Individual Participant Data (IPD) NMA model can be fit, including estimating TCIs. A key aspect of model specification in IPD NMA is the choice to use a one-stage or two-stage model. In a one-stage model, all data are synthesized simultaneously in a single model, whereas in a two-stage model, study-level estimates of relative effects and interactions are determined separately in stage one and then synthesized in a NMA model in stage two [5, 6]. Care must be taken in the specification of one-stage models to ensure that within-trial information is not mixed with across-trial information as this can lead to the same ecological bias and confounding present in the NMA meta-regression model [1, 6, 7, 5]. Personalized treatment hierarchies can be produced from IPD NMA when it is certain that a two-stage model or a carefully specified one-stage model is used.

NMA models were recently developed to estimate Individualized Treatment Rules (ITRs) [8]. An ITR is a rule that takes patient characteristics as input and outputs the expected optimal treatment [9]. ITR NMA is similar to an IPD NMA in the data it requires and the estimates it provides. When using ITR NMA, additional assumptions are made that are common in the causal literature, to ensure that the relative treatment effects have causal interpretations [8, 9]. The assumptions are: i) The Stable Unit Treatment Value Assumption (SUTVA, meaning participants do not influence each other's responses), ii) there are no unmeasured confounders, iii) positivity, meaning that there is a positive probability of receiving every possible combination of treatment and covariate values among patients in the population. Additionally, for the approach described in [8], the outcome model must be correctly specified. The existing ITR NMA approach is a two-stage model, preventing across-trial information from biasing TCI estimation. When the assumptions are satisfied, ITR NMA models are a promising approach to creating personalized treatment hierarchies.

S2 Description of the MDD Study Datasets

The included studies (STAR*D, EMBARC, and REVAMP) are multistage randomized trials, and the details of their designs can be found in [10], [11], and [12] respectively. We focus on pharmacological treatments only. Patients who had missing covariate or outcome data were removed from the analysis. The number of patients receiving each treatment in each study is shown in Table S1. To ensure reliable estimates of study-level TCIs and variances, we

exclude patients whose treatment group had fewer than 20 participants in the study. The set of treatments in the network is Bupropion (BUP), Citalopram and Buproprion (CIT + BUP), Citalopram + Buspirone (CIT+BUS), Escitalopram (ESCIT), Sertraline (SER) and Venlafaxine (VEN). A network plot of the evidence is shown in Figure S1.

Table S1: Number of patients who received each pharmacological treatment considered in the analysis in each study. *Not included in the analysis due to the sample size being below 20.

Study	SER	BUP	CIT + BUP	CIT+BUS	ESCIT	VEN
STAR*D	74	82	97	121	0	72
EMBARC	73	49	0	0	0	0
REVAMP	252	27	0	0	36	11*

STAR*D is a four-stage study. Patients were all assigned to the same treatment, Citalopram, in stage one. In stages three and four, sample sizes are limited. Thus, we use data only from stage two of this study, representing patients who did not respond to Citalopram in stage one, and were randomized to other treatments in stage two. In the EMBARC study, patients received placebo or SER in stage one, and non-responders received either BUP or SER in stage two. We include only stage two in our analysis. We use data from the first stage of REVAMP, where an algorithm was used to assign patients to treatments. The population represented by the network of evidence is thus patients with MDD who may not be treatment-naive.

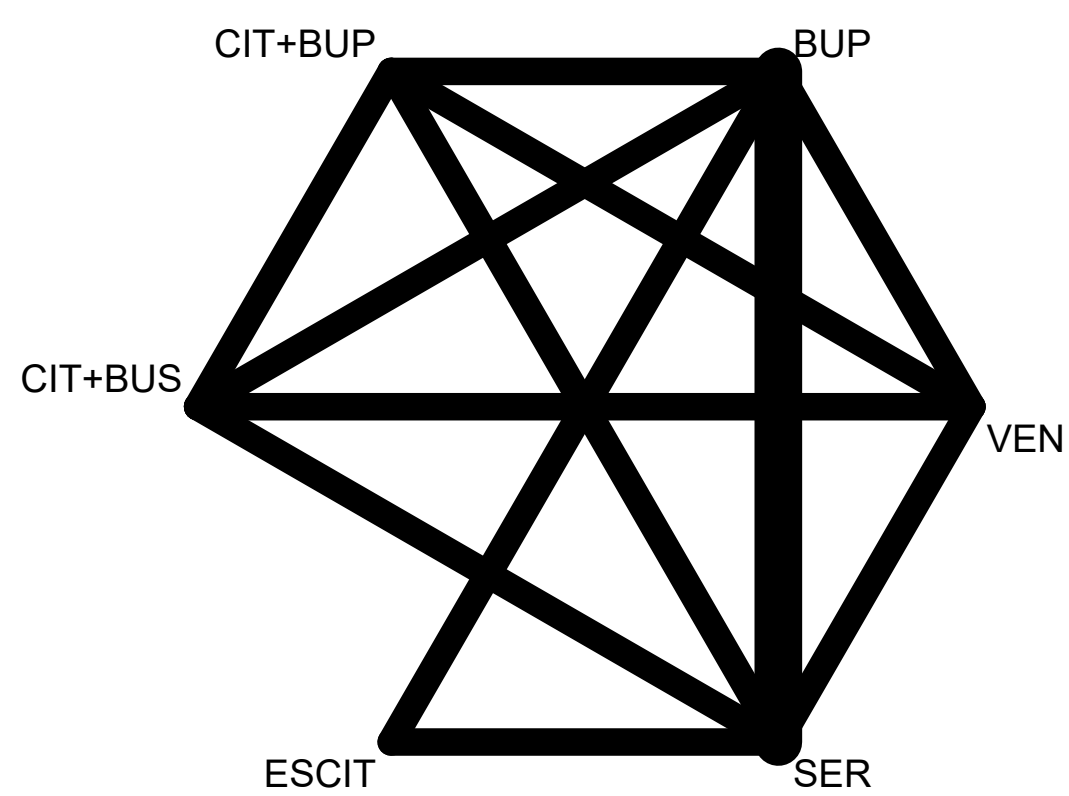

Figure S1: A network plot of the MDD studies. The line thickness is proportional to the number of studies making the direct comparison in the data. All direct comparisons are observed in only one study with the exception of SER vs. BUP, which occurs in all three studies.

S3 ITR NMA Model and Analysis

ITR NMA was introduced by [8]. As discussed in Section S1.2, ITR NMA provides estimates of relative treatment effects and treatment-covariate interactions and requires additional assumptions that are common in the causal literature to ensure that the relative treatment effects have causal interpretations. We used a two-stage ITR NMA model similar to that described in [8] to analyse the MDD data. We used a common effect model that did not

estimate between-study heterogeneity because there was limited repeated pairwise comparisons in the data as shown in Figure S1, making it difficult to estimate heterogeneity.

S3.1 Model Description

Suppose we have I studies indexed by i, which together form a network of evidence for G treatments indexed by $g = 1, \ldots, G$. Each study compares a subset of $G_i \leq G$ treatments indexed by t_{ik}, $k = 1, \ldots, G_i$, so that t_{ik} is the index of the kth treatment in study i. For ease of presentation, we assume that treatment 1 is employed in arm 1 of each study, that is, $t_{i1} = 1$ for $i = 1, \ldots, I$, so that the reference treatment in the study-level models is the same as the reference treatment in the NMA model.

The study-level data is represented by the response of patient j in study i, Y_{ij}, a vector of the covariates recorded for patient j in study i, $\boldsymbol{x_{ij}}$, and $\boldsymbol{a_{ij}} = (a_{ijt_{i2}}, \ldots, a_{ijt_{iG_i}})^T$, a vector encoding the treatment received by patient ij, where $a_{ijt_{ik}} = 1$ if patient j received the kth treatment in study i (or equivalently, they received treatment t_{ik}), and 0 otherwise. The response is the negative of the HRSD-17. In the first stage, study-specific estimates of relative treatment effects for the treatments observed in the study are obtained by fitting a linear model to the data. The linear model is of the form

$$\mathrm{E}(Y_{ij} \mid \boldsymbol{X_{ij}} = \boldsymbol{x_{ij}}, \boldsymbol{A_{ij}} = \boldsymbol{a_{ij}}) = \boldsymbol{\beta_i}^T \boldsymbol{x_{ij}} + \boldsymbol{a_{ij}}^T \underline{\boldsymbol{\delta_i}^{(1)}} \boldsymbol{x_{ij}^{(\delta)}}, \tag{1}$$

where $\boldsymbol{x_{ij}}$ contains P covariates, which should include prognostic and effect-modifying variables and a leading 1, $\boldsymbol{\beta_i}$ is a $P+1$ dimensional vector encoding the effect of covariates for patients receiving treatment 1, $\boldsymbol{x_{ij}^{(\delta)}}$ is a subvector of $\boldsymbol{x_{ij}}$ containing Q effect-modifying variables, and $\underline{\boldsymbol{\delta_i}^{(1)}}$ is a $(G_i - 1) \times (Q + 1)$ matrix encoding relative treatment effects and TCIs. More specifically,

$$\underline{\boldsymbol{\delta_i}^{(1)}} = \begin{pmatrix} \delta^{(1)}_{i,t_{i2}0} & \delta^{(1)}_{i,t_{i2}1} & \cdots & \delta^{(1)}_{i,t_{i2}Q} \\ \delta^{(1)}_{i,t_{i3}0} & \delta^{(1)}_{i,t_{i3}1} & \cdots & \delta^{(1)}_{i,t_{i3}Q} \\ \vdots & \vdots & \ddots & \vdots \\ \delta^{(1)}_{i,t_{iG_i}0} & \delta^{(1)}_{i,t_{iG_i}1} & \cdots & \delta^{(1)}_{i,t_{iG_i}Q} \end{pmatrix}, \tag{2}$$

where $\delta^{(1)}_{i,g0}$ is the true study-specific relative effect of treatment g compared to treatment 1 and $\delta^{(1)}_{i,gq}, q > 0$ is the true study-specific interaction of covariate q with treatment g compared to treatment 1. Note that equation (1) is linear in parameters, but non-linear transformations of covariates can be included (e.g., polynomials or splines) to ensure the assumption of correct model specification is met.

The first term of equation (1), $\boldsymbol{\beta_i}^T \boldsymbol{x_{ij}}$, is sometimes called the treatment-free model, but in the context of NMA, we will call it the reference treatment model, as it represents the impact of the covariates on patients who received the reference treatment. For this reason, all prognostic and effect-modifying variables should be included in $\boldsymbol{x_{ij}}$. The term $\boldsymbol{a_{ij}}^T \underline{\boldsymbol{\delta_i}^{(1)}} \boldsymbol{x_{ij}^{(\delta)}}$ is called the blip model, and only effect-modifying covariates need be included in $\boldsymbol{x_{ij}^{(\delta)}}$. Estimates of the reference treatment model parameters, as well as the blip parameters and their corresponding variance-covariance matrix are obtained using ordinary least squares. The reference treatment model parameters are not synthesized in the second stage of the model, but correct specification of this part of the model is required for consistent estimation of the $\underline{\boldsymbol{\delta_i}^{(1)}}$ parameters [9].

For the second stage of the model, we introduce $\hat{\boldsymbol{\delta}}_{\boldsymbol{i}}^{(1)}$, a vector of length $(G_i - 1) \times (Q + 1)$ that stores the blip parameter estimates from the linear model. Let $\boldsymbol{S_i}$ be the estimated variance-covariance matrix of $\hat{\boldsymbol{\delta}}_{\boldsymbol{i}}^{(1)}$ obtained from the ordinary least squares model fit. Note that this differs from the approach taken by [8], as their model assumes that if $q \neq q'$, then $Cov(\hat{\delta}^{(1)}_{i,kq}, \hat{\delta}^{(1)}_{i,lq'}) = 0$ for any k, l so that $\boldsymbol{S_i}$ is block-diagonal. Let $\boldsymbol{\psi}^{(1)}$ be a vector of the $(G - 1) \times (Q + 1)$ parameters that characterize the true pooled relative treatment effects and TCIs, and let $\boldsymbol{V_i}$ be a $[(G_i - 1) \times (Q + 1)] \times [(G - 1) \times (Q + 1)]$ design matrix that maps the study-specific parameters to their pooled counterparts, so that $\boldsymbol{V}_{\boldsymbol{i}nm} = 1$ if the nth element of $\hat{\boldsymbol{\delta}}_{\boldsymbol{i}}^{(1)}$ contributes to estimating the mth element of $\boldsymbol{\psi}^{(1)}$ and zero otherwise. $\boldsymbol{V_i}$ is needed because each study investigates a different subset of treatments, and thus each $\hat{\boldsymbol{\delta}}_{\boldsymbol{i}}^{(1)}$ only directly contributes to estimating a subset of $\boldsymbol{\psi}^{(1)}$ unless the study compared all treatments in the network. The first $G - 1$ parameters in $\boldsymbol{\psi}^{(1)}$ correspond to the true pooled relative treatment effects compared to treatment

1, that is, $\psi^{(1)}_{g0}, g = 2, \ldots, G$. The following block of $G-1$ parameters corresponds to the interaction of treatments $2, \ldots, G$ with covariate 1, the next block of $G-1$ parameters with covariate 2, and so on.

The common-effect second stage model is given by

$$\hat{\boldsymbol{\delta}}_{\boldsymbol{i}}^{(1)} \sim \text{MVN}(\boldsymbol{V_i}\boldsymbol{\psi}^{(1)}, \boldsymbol{S_i}) \quad i = 1, \ldots, I \tag{3}$$

$$\boldsymbol{\psi}^{(1)} \sim \text{MVN}(\boldsymbol{0}, 100^2\boldsymbol{\mathcal{I}}), \tag{4}$$

where $\boldsymbol{0}$ is a length $(G-1) \times (Q+1)$ vector of zeroes and $\boldsymbol{\mathcal{I}}$ is an identity matrix of dimension $[(G-1) \times (Q+1)] \times [(G-1) \times (Q+1)]$. We fit the model to the study-level parameter estimates using Markov Chain Monte Carlo (MCMC) sampling via Stan in R through the `rstan` package [13, 14]. We ran four independent chains with 2500 warm-up iterations and 2500 burn-in iterations. Convergence was assessed by inspecting traceplots and ensuring that the potential scale reduction factor, $\hat{R}$, was less than 1.005 for all parameters [15, 13]. This model could be extended to allow for random-effects and studies which do not include treatment 1.

To perform personalized inference, we also define the unstacked version of $\boldsymbol{\psi}^{(1)}$, $\underline{\boldsymbol{\psi}^{(1)}}$, which is a matrix of dimension $(G-1) \times (Q+1)$ similar to the definition of $\underline{\boldsymbol{\delta}_{\boldsymbol{i}}^{(1)}}$, but with population-level rather than study-level parameters, such that

$$\underline{\boldsymbol{\psi}^{(1)}} = \begin{pmatrix} \psi^{(1)}_{20} & \psi^{(1)}_{21} & \cdots & \psi^{(1)}_{2Q} \\ \psi^{(1)}_{30} & \psi^{(1)}_{31} & \cdots & \psi^{(1)}_{3Q} \\ \vdots & \vdots & \ddots & \vdots \\ \psi^{(1)}_{G0} & \psi^{(1)}_{G1} & \cdots & \psi^{(1)}_{GQ} \end{pmatrix}, \tag{5}$$

noting that the first column corresponds to the population-level relative effects when all covariates are zero as described in the main text.

Letting $\boldsymbol{a}$ be a vector of length $G-1$ coding the treatment assignment and $\boldsymbol{x}$, a vector of length $Q+1$ coding the covariate profile of interest (and a leading 1). The expected outcome for a particular covariate profile for the treatment a, coded by $\boldsymbol{a}$, versus treatment 1 is given by

$$\boldsymbol{a}^T \underline{\boldsymbol{\psi}^{(1)}} \boldsymbol{x} = \psi^{(1)}_{a0} + \psi^{(1)}_{a1}x_1 + \psi^{(1)}_{a2}x_2 + \cdots + \psi^{(1)}_{aQ}x_Q. \tag{6}$$

The posterior distribution of $\boldsymbol{a}^T \underline{\boldsymbol{\psi}^{(1)}} \boldsymbol{x}$ can be obtained by computing it for every posterior sample of $\underline{\boldsymbol{\psi}^{(1)}}$.

S3.2 Variable Definitions and Covariate Profiles

The covariates included in the reference treatment model were the patient's age, sex, race, marital status, years of education completed, employment status, household size, age at onset of MDD, number of MDD episodes, chronic status of current episode, and baseline HRSD-17 before receiving any treatments in their respective study. The covariates included in the blip model are the same as those in the reference treatment model, with the exception of race, as it is not recommended to tailor treatments based on race [8]. Continuous variables were centred so that the interpretation of the covariate reference level (ie, when the covariate is equal to zero) would not require extrapolation outside the range of the data. Variable definitions are summarized in Table S2. Due to the multicategory definition of marital status, there are $P = 12$ covariates total and $Q = 11$ tailoring variables.

S3.3 Covariate Settings for Personalized Treatment Hierarchies

The covariate profiles of Patient A and Patient B were the same for all variables except sex, employment status, household size, and number of MDD episodes. All other blip model covariates were set to 0, corresponding to the reference levels described in Table S2. The settings and interpretation of the covariates that differed between patients is described in Table S3. Note that only the covariates in the blip model need to be specified to make a personalized treatment hierarchy.

S3.4 Additional Results

A plot of the posterior means and 95% credible intervals for all blip model parameters is shown in Figure S2.

Table S2: Variable definitions in the MDD ITR NMA.

Covariate	Definition in Model	Interpretation of reference level	Model(s)
Age	Age in years minus 40	Age 40	$\boldsymbol{x_{ij}}$, $\boldsymbol{x_{ij}^{(\delta)}}$
Sex	Sex reported by patient, coded as one binary variable that equals 1 if the patient reported Male and 0 otherwise	Female or other sex	$\boldsymbol{x_{ij}}$, $\boldsymbol{x_{ij}^{(\delta)}}$
Race	Race reported by patient, coded as one binary variable that equals 1 if the patient reported White and 0 otherwise	Race other than White	$\boldsymbol{x_{ij}}$
Marital status	Marital status of patient: married, single, or divorced/widowed. Coded using two binary variables; one that equals 1 if the patient was single, and another that equals 1 if the patient was divorced/widowed.	Married	$\boldsymbol{x_{ij}}$, $\boldsymbol{x_{ij}^{(\delta)}}$
Years of education	Years of formal education completed by the patient minus 14	14 years of education completed	$\boldsymbol{x_{ij}}$, $\boldsymbol{x_{ij}^{(\delta)}}$
Employment status	Employment status reported by patient: employed or unemployed/retired, coded as a single binary variable that equals 1 if the patient was unemployed/retired and 0 otherwise	Employed	$\boldsymbol{x_{ij}}$, $\boldsymbol{x_{ij}^{(\delta)}}$
Household size	The number of people living in the patient's household, including the patient, minus 3	Household size of 3	$\boldsymbol{x_{ij}}$, $\boldsymbol{x_{ij}^{(\delta)}}$
Age at onset of MDD	Age at onset of MDD in years minus 18	Onset of MDD at age 18	$\boldsymbol{x_{ij}}$, $\boldsymbol{x_{ij}^{(\delta)}}$
Number of MDD episodes	Number of MDD episodes experienced by the patient in their lifetime, coded as a binary variable that equals 1 if the patient had more than 3 episodes and 0 otherwise [8]	Three or fewer MDD episodes in their lifetime	$\boldsymbol{x_{ij}}$, $\boldsymbol{x_{ij}^{(\delta)}}$
Chronic status of episode	Chronic status of current MDD episode, coded as a binary variable that equals one if the current episode has lasted for 2 years or more and 0 otherwise	Current episode is not chronic (has lasted less than 2 years)	$\boldsymbol{x_{ij}}$, $\boldsymbol{x_{ij}^{(\delta)}}$
Baseline HRSD-17	HRSD-17 at intake to the study minus 22	HRSD-17 at baseline of 22	$\boldsymbol{x_{ij}}$, $\boldsymbol{x_{ij}^{(\delta)}}$

Table S3: Covariate values and their interpretation for the covariates that differed between Patient A and Patient B. All other covariate values are set to 0.

Variable	Patient A		Patient B	
	Value	Interpretation	Value	Interpretation
Sex	1	Male	0	Female
Employment status	0	Employed	1	Unemployed or retired
Household size	1	4 household members	-1	2 household members
Number of episodes	0	≤ 3 episodes	1	> 3 episodes

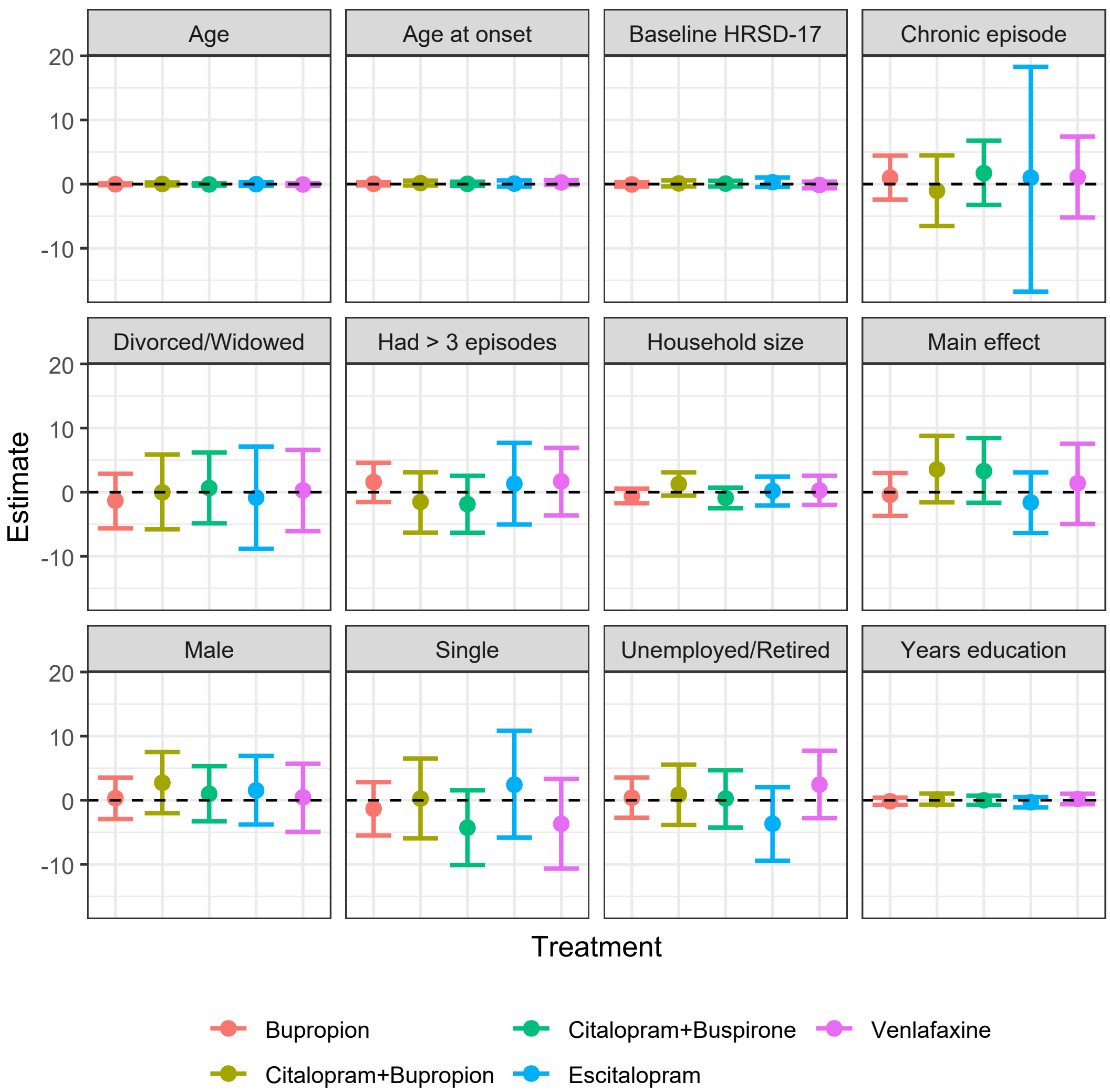

Figure S2: Point estimates and 95% credible intervals for $\psi^{(1)}$ from the ITR NMA model of MDD data.

References

1. Donegan S, Williamson P, D'alessandro U, Smith CT. Assessing the Consistency Assumption by Exploring Treatment by Covariate Interactions in Mixed Treatment Comparison Meta-Analysis: Individual Patient-Level Covariates versus Aggregate Trial-Level Covariates. *Statistics in Medicine*. 2012; 31(29):3840–3857.
2. Riley RD, Lambert PC, Staessen JA, et al. Meta-Analysis of Continuous Outcomes Combining Individual Patient Data and Aggregate Data. *Statistics in Medicine*. 2008; 27(11):1870–1893.
3. Berlin JA, Santanna J, Schmid CH, Szczech LA, Feldman HI. Individual Patient- versus Group-Level Data Meta-Regressions for the Investigation of Treatment Effect Modifiers: Ecological Bias Rears Its Ugly Head. *Statistics in Medicine*. 2002; 21(3):371–387.
4. Schmid CH, Stark PC, Berlin JA, Landais P, Lau J. Meta-Regression Detected Associations between Heterogeneous Treatment Effects and Study-Level, but Not Patient-Level, Factors. *Journal of Clinical Epidemiology*. 2004; 57(7):683–697.
5. Riley RD, Dias S, Donegan S, et al. Using Individual Participant Data to Improve Network Meta-Analysis Projects. *BMJ Evidence-Based Medicine*. 2023; 28(3):197–203. PMID: 35948411.
6. Hua H, Burke DL, Crowther MJ, Ensor J, Tudur smith C, Riley RD. One-Stage Individual Participant Data Meta-Analysis Models: Estimation of Treatment-Covariate Interactions Must Avoid Ecological Bias by Separating out {W}ithin-Trial and {A}cross-Trial Information. *Statistics in Medicine*. 2017; 36(5):772–789.
7. Riley RD, Debray TP, Fisher D, et al. Individual Participant Data Meta-Analysis to Examine Interactions between Treatment Effect and Participant-Level Covariates: Statistical Recommendations for Conduct and Planning. *Statistics in Medicine*. 2020; 39(15):2115–2137.
8. Shen J, Moodie EE, Golchi S. Two-Stage Bayesian Network Meta-Analysis of Individualized Treatment Rules for Multiple Treatments with Siloed Data. *Statistical Methods in Medical Research*. 2025; 35(1):3–20.
9. Tsiatis AA, Davidian M, Holloway ST, Laber EB. *Dynamic Treatment Regimes: Statistical Methods for Precision Medicine*. New York: Chapman and Hall/CRC, Dec. 18, 2019. 618 pp.
10. Rush AJ, Fava M, Wisniewski SR, et al. Sequenced Treatment Alternatives to Relieve Depression (STAR*D): Rationale and Design. *Controlled Clinical Trials*. 2004; 25(1):119–142.
11. Trivedi MH, Mcgrath PJ, Fava M, et al. Establishing Moderators and Biosignatures of Antidepressant Response in Clinical Care (EMBARC): Rationale and Design. *Journal of Psychiatric Research*. 2016; 78():11–23.
12. Trivedi MH, Kocsis JH, Thase ME, et al. REVAMP - Research Evaluating the Value of Augmenting Medication with Psychotherapy: Rationale and Design. *Psychopharmacology Bulletin*. 2008; 41(4):5–33. PMID: 19015627.
13. Stan Development Team. *RStan: The R Interface to Stan*. Version 2.32.6. 2024.
14. Stan Development Team. *Stan User's Guide*. Version 2.37. 2025.
15. Vehtari A, Gelman A, Simpson D, Carpenter B, Bürkner PC. Rank-Normalization, Folding, and Localization: An Improved Rhat for Assessing Convergence of MCMC (with Discussion). *Bayesian Analysis*. 2021; 16(2):667–718.
